# Comprehensive Laboratory Evaluation of a Lateral Flow Assay for the Detection of *Yersinia pestis*

**DOI:** 10.1089/hs.2019.0094

**Published:** 2019-12-16

**Authors:** Kristin W. Prentice, Lindsay DePalma, Jason G. Ramage, Jawad Sarwar, Nishanth Parameswaran, Jeannine Petersen, Brook Yockey, John Young, Mrinmayi Joshi, Nagarajan Thirunavvukarasu, Ajay Singh, Carol Chapman, Julie R. Avila, Christine A. Pillai, Gowri Manickam, Shashi K. Sharma, Stephen A. Morse, Kodumudi Venkat Venkateswaran, Kevin Anderson, David R. Hodge, Segaran P. Pillai

**Affiliations:** Kristin W. Prentice, MS, is an Associate, Booz Allen Hamilton, Rockville, MD. Lindsay DePalma, MS, is a Staff Life Scientist, Booz Allen Hamilton, McLean, VA. Jason G. Ramage, MS, MBA, PMP, is Assistant Vice Chancellor for Research and Innovation and Director of Research Compliance, University of Arkansas, Fayetteville, AR. Jawad Sarwar, MS, is a Senior Research Scientist, and Nishanth Parameswaran and Mrinmayi Joshi, MS, are Research Scientists; all at Omni Array Biotechnology, Rockville, MD. Jeannine Petersen, PhD, Brook Yockey, and John Young are Microbiologists; all with DHHS/CDC/OID/NCEZID/DVBD/BDB, Fort Collins, CO. Nagarajan Thirunavvukarasu, PhD, is an ORISE Fellow; Christine A. Pillai and Gowri Manickam, PhD, are ORISE Fellow Research Scientists; and Shashi K. Sharma, PhD, is a Research Microbiologist; all at the FDA Center for Food Safety and Applied Nutrition, Molecular Methods Development Branch, Division of Microbiology, Office of Regulatory Science, College Park, MD. Ajay Singh, PhD, is a Research Scientist, Laulima Government Solutions, Contractor Support to USAMRICD, Neurobiological Toxicology Branch, Analytical Toxicology Division, Aberdeen Proving Ground, MD. Carol Chapman, MS, is a Microbiologist, Geneva Foundation, Contractor Support to the Naval Medical Research Center, Silver Spring, MD. Julie R. Avila, MS, is a Scientific Associate, Lawrence Livermore National Laboratory, Biosciences and Biotechnology Division, Livermore, CA. Stephen A. Morse, MSPH, PhD, is a Senior Advisor, CDC Division of Select Agents and Toxins, and is currently with IHRC, Inc., Atlanta, GA. Kodumudi Venkat Venkateswaran, PhD, is Chief Scientist, Tetracore, Inc., Rockville, MD. Kevin Anderson, PhD, and David R. Hodge, PhD, are Program Managers, Science and Technology Directorate, US Department of Homeland Security, Washington, DC. Segaran P. Pillai, PhD, is Director, Office of Laboratory Science and Safety, FDA Office of the Commissioner, Silver Spring, MD.

**Keywords:** *Yersinia pestis, Plague*, Lateral flow immunoassay, Rapid detection

## Abstract

We conducted a comprehensive, multiphase laboratory evaluation of the Plague BioThreat Alert^®^ (BTA) test, a lateral flow immunoassay (LFA), for the rapid detection of *Yersinia pestis*. The study was conducted in 7 phases at 2 sites to assess the performance of the LFA. The limit of detection (LOD) was determined using both a virulent and avirulent strain of *Y. pestis*, CO99-3015 (10^5^ CFU/ml) and A1122 (10^4^ CFU/ml), respectively. In the other phases, 18 *Y. pestis* strains, 20 phylogenetic near-neighbor strains, 61 environmental background microorganisms, 26 white powders, and a pooled aerosol sample were also tested. A total of 1,110 LFA test results were obtained, and their analysis indicates that this LFA had a sensitivity of 97.65% and specificity of 96.57%. These performance data are important for accurate interpretation of qualitative results arising from testing suspicious white powders and aerosol samples in the field. Any positive specimen in this assay is considered presumptive positive and should be referred to the Centers for Disease Control and Prevention Laboratory Response Network for additional testing, confirmation, and characterization for an appropriate public health response.

## Abstract 2

The authors conducted a comprehensive, multiphase laboratory evaluation of the Plague Bio Threat Alert® (BTA) test, a lateral flow immunoassay (LFA), for the rapid detection of *Yersinia pestis*. A total of 1,110 LFA test results were obtained, and their analysis indicates that this LFA had a sensitivity of 97.65% and specificity of 96.57%. These performance data are important for accurate interpretation of qualitative results arising from testing suspicious white powders and aerosol samples in the field.

Y*ersinia pestis* is the causative agent of plague. It is a Gram-negative, nonmotile, non–spore forming coccobacillus, which is urease and indole negative.^1-7^ As a facultative anaerobe belonging to the Yersiniaceae, *Y. pestis* evolved from *Yersinia pseudotuberculosis* between 2,600 and 28,000 years ago, after the acquisition of 2 *Y. pestis*–specific plasmids: pMT1 and pPCP1.^8,9^ *Y. pestis* grows at 28°C, which is the normal body temperature for fleas, and at 37°C, the normal body temperature for humans.^1,4,6,7^ In the laboratory, the bacterium can grow on standard microbiologic media.^4^ Observable growth is present at 24 to 48 hours, although colonies are smaller than those seen with other bacteria.^4,7^ In the wild, *Y. pestis* infects rodents, including rats, squirrels, and prairie dogs.^1,4,10,11^ Infection usually results from being bitten with a *Y. pestis*–infected vector such as the Oriental rat flea (*Xenopsylla cheopis*).^1,3,4,10,12-15^ Less commonly, infection can occur through handling infected animals or from inhaling infective respiratory droplets or aerosols.^3^

All 3 pathogenic species of *Yersinia* (*Y. pestis*, *Y. pseudotuberculosis,* and *Yersinia enterocolitica*) carry the 70 kbp pCD1 plasmid, which encodes for a type III secretion system and *Yersinia* outer membrane proteins (Yops) that enable these bacteria to evade the host immune system. The pCD1 plasmid is not found in nonpathogenic *Yersinia* species.^9,13,16-18^ Virulent strains of *Y. pestis* also possess 2 additional plasmids: the 90 kbp pMT1 plasmid, alternatively known as pFra, which encodes the F1 capsular protein, and murine toxin.^19^ The F1 capsule enables *Y. pestis* to resist engulfment by neutrophils and macrophages.^1,12^ The F1 gene is temperature regulated and expressed at ≥33°C. The 9.5 kbp pPCP1 plasmid, also known as pPla or pPst, encodes for plasminogen-activating factor as well as pestin and coagulase.^10,19^ In the laboratory, the plasmids can be lost during storage or on subculture.^19,20^

There are 3 clinical forms of plague: bubonic, septicemic, and pneumonic. The most common form is bubonic plague, acquired through the bite of an infected flea, which accounts for approximately 80% to 95% of all cases worldwide. Infected humans develop regional lymph node swelling and fever; bubonic plague is not transmissible from person to person and has a mortality rate of approximately 50% if untreated. Following entry into the body, *Y. pestis* is phagocytized by both neutrophils and macrophages; however, the bacteria survive and replicate in macrophages. Following infiltration of the lymph nodes, bacteria may enter the bloodstream, resulting in septicemic plague, which can also be caused directly via wound exposure.^4-6,14,15,21-23^

Pneumonic plague is far more dangerous but is very rare. Secondary pneumonic plague may result from the dissemination of bacteria to the lungs in cases of bubonic or septicemic plague, leading to severe bronchopneumonia, chest pain, dyspnea, cough, and hemoptysis. Primary pneumonic plague results from the direct inhalation of airborne droplets or aerosols of *Y. pestis* and is the clinical form most likely to occur following an aerosol release in a bioterrorism attack. Pneumonic plague is transmissible from person to person via airborne droplets. Infected individuals experience a 2- to 4-day incubation period, followed by rapid onset of chills, fever, general malaise, increased heart and respiratory rates, elevated body temperature, and a cough that becomes bloody as the disease progresses. During the terminal stage of the disease, patients experience hemorrhagic necrosis, acute respiratory failure, sepsis, and circulatory collapse. Diagnosis is based on immunostaining, PCR, and culture. In the absence of prompt treatment with antibiotics, pneumonic plague has a mortality rate that approaches 100%. Recommended treatment involves a 10- to 14-day regimen with an antibiotic such as streptomycin, gentamicin, ciprofloxacin, levofloxacin, or doxycycline.

First responders often encounter unknown and suspicious white powders in the field, and it is important to quickly evaluate them for the possible presence and identification of biological threat agents. The results of these evaluations can inform decisions to initiate public safety actions, including area evacuation, facility closure to prevent additional exposures, decontamination and initiation of medical countermeasures for potentially exposed individuals, collection of samples for law enforcement and public health purposes, sample transfer to a reference laboratory for immediate testing, and containment of contamination to prevent secondary dissemination or reaerosolization. In order to support first responders with the appropriate tools to carry out their mission, there is a need to understand performance of rapid assays for screening suspicious white powders.

The purpose of the present study was to evaluate the sensitivity, specificity, limit of detection (LOD), reproducibility, and limitations of the Plague BioThreat Alert test (Tetracore, Inc., Rockville, MD) as pertains to its use in the field to screen for the presence of *Y. pestis*. The goal of this study was to provide an understanding of assay performance, including the likelihood of false-negative results (assay is negative, but the target analyte is present at a concentration at or above the LOD), false-positive results (assay is positive, but the target analyte is not present in the sample), the limit of detection, and reproducibility, so that appropriate and effective decisions can be made by first responders to support public safety actions while avoiding unnecessary fear, panic, and costly civil disruptions.

This study was designed and executed through an interagency collaboration with participation from subject matter experts from the Department of Homeland Security (DHS), the Department of Health and Human Services (HHS), the Department of Justice (DOJ), the US Department of Agriculture (USDA), and the US Secret Service (USSS).

## Materials and Methods

### Biosafety Considerations

Strains used in this study were handled with appropriate biosafety conditions in accordance with the 5th edition of *Biosafety in Microbiological and Biomedical Laboratories* (BMBL)^24^ and the Federal Select Agent Regulations.

### Plague BTA Test and BioThreat Alert Reader MX

The Plague BTA test, which employs antibodies to detect the presence of the F1 capsular antigen, BioThreat Alert Reader MX (BTA Reader), and BTA Sample Buffer (BTA buffer) were obtained from Tetracore, Inc. (Rockville, MD). The performance of the Plague LFA and BTA Reader was evaluated at 2 test sites: (1) the Centers for Disease Control and Prevention (CDC), Fort Collins, CO, and (2) Omni Array Biotechnology Corporation, Rockville, MD. Samples for phases 1, 2, and 7 were prepared and tested at CDC, and samples for phases 3 to 6 were prepared and stored at 4°C at Omni Array until use, then analyzed by personnel from DHS Science and Technology (S&T) and FDA Center for Food Safety and Applied Nutrition (CFSAN), according to a standard protocol provided by the manufacturer.

Plague BTA LFA results were read both visually and with a BTA Reader, according to the directions provided by the manufacturer—that is, between 15 and 30 minutes after adding the sample (0.150 mL) to the BTA LFA. Samples with readings of <200 were considered negative, and test strips that failed to develop a control line were noted and discarded. Positive or negative determinations were based on the reader result. BTA buffer was used as a negative control. *Y. pestis* strain A1122 (avirulent, select agent exempt strain) was used as a positive control at a concentration 2 logs above LOD (10^7^ to 10^8^) at both test sites.

### Culture Preparation

All bacterial isolates used in phases 1 and 2 were grown and prepared at CDC. Isolates were inoculated onto 6% sheep blood agar (SBA) and incubated at 35°C for 24 hours. Isolates were subcultured to confirm purity and then used to prepare cell suspensions. Suspensions were prepared in 2 mL of 0.85% sterile saline and lightly vortexed. Stock suspensions were adjusted to an OD600 absorbance of 1.0 (5.5 x 10^9^ CFU/mL) using a Microscan turbidity meter (Dade Behring, Inc., Deerfield, IL). The CFU/mL for a *Y. pestis* cell suspension with an OD600 of 1.0 was determined by colony counts, and this absorbance was subsequently used for preparing for cell suspensions of assigned concentrations.

### Phase 1: Linear Dynamic Range and Repeatability Study

The linear dynamic range of the Plague LFA was determined using both the virulent (CO99-3015) and avirulent (A1122) strains of *Y. pestis*. Bacterial suspensions for testing were prepared by diluting the stock suspension 10-fold dilution in BTA buffer to achieve concentrations of 10^8^-10^9^ CFU/mL, 10^7^-10^8^ CFU/mL, 10^6^-10^7^ CFU/mL, 10^5^-10^6^ CFU/mL, 10^4^-10^5^ CFU/mL, 10^3^-10^4^ CFU/mL, and 10^2^-10^3^ CFU/mL. Each dilution was quantified by plating 0.1 mL dilutions in triplicate onto SBA agar plates and counting colonies after 48 hours' incubation at 35°C. For testing, each suspension was lightly vortexed and immediately tested by adding 0.150 mL to the sample well of a test strip. Each concentration was tested 5 times, and the results read both visually and with 1 of 2 BTA Readers. The lowest concentration that yielded positive results in 5 out of 5 tests was further evaluated with an additional 120 test strips (repeatability study), with results read both visually and with 1 of 2 BTA Readers.

### Phase 2: Inclusivity Panel

In order to determine whether the Plague LFA assay could detect diverse strains of *Y. pestis*, a total of 18 *Y. pestis* strains (Table 1) were grown at 35°C for 24 hours. Bacterial suspensions were prepared and diluted in BTA buffer to a final concentration of 10^7^ to 10^8^ CFU/mL (2 logs above the LOD) and vortexed, and a 0.150-mL volume was added to the sample port of 5 Plague BTA test strips. Results were read both visually and with 1 of 2 BTA Readers.

**Table 1.** *Y. pestis* inclusivity strains

S. No.	Species	Strain ID
1	*Yersinia pestis biovar* antiqua	UG05-0454
2	*Yersinia pestis biovar* antiqua	Antiqua
3	*Yersinia pestis biovar* antiqua	Nepal 516
4	*Yersinia pestis biovar* mediaevalis	Nicholisk 51
5	*Yersinia pestis biovar* mediaevalis	KIM 10
6	*Yersinia pestis biovar* mediaevalis	PyH1R3
7	*Yersinia pestis biovar* mediaevalis	PKH-10
8	*Yersinia pestis biovar* orientalis	CA00-2641
9	*Yersinia pestis biovar* orientalis	AZ94-0666
10	*Yersinia pestis biovar* orientalis	NM99-0030
11	*Yersinia pestis biovar* orientalis	ZE94-2122
12	*Yersinia pestis biovar* orientalis	P. Exu 2
13	*Yersinia pestis biovar* orientalis	A1122
14	*Yersinia pestis biovar* orientalis	EV 76
15	*Yersinia pestis biovar* orientalis	CO99-3015 OR CO92
16	*Yersinia pestis biovar* mediaevalis	Pestoides A
17	*Yersinia pestis biovar* antiqua	Pestoides F
18	*Yersinia pestis biovar* antiqua	Angola

### Phase 3: Near Neighbor Panel

Suspensions of 20 phylogenetic near neighbors of *Y. pestis* (Table 2) were prepared and diluted in BTA buffer to a concentration of 10^8^ to 10^9^ CFU/mL (3 logs above the LOD). After vortexing, a 0.150-mL volume of each suspension was added to the sample wells of 5 test strips. Results were read both visually and with 2 BTA Readers.

**Table 2.** *Y. pestis* Near Neighbor Panel

S. No.	Strain Name	Other Identifier	Biovar/ Serotype	Genome Homology
1	*Yersinia enterocolitica*	ATCC 9610	0:8; Biovar 1	98%
2	*Yersinia enterocolitica*	ATCC 700823	0:9: Biovar 2	
3	*Yersinia enterocolitica*	ATCC 700822	0:3; Biovar 4	
4	*Yersinia enterocolitica* subs. *palearctica*	DSM 13030		
5	*Yersinia entomophaga*	DSM 22339		
6	*Yersinia massiliensis*	DSM 21859		
7	*Yersinia nurmii*	DSM 22296		
8	*Yersinia pekkanenii*	DSM 22769		
9	*Yersinia pseudotuberculosis*	ATCC 6902	1a	99%
10	*Yersinia pseudotuberculosis*	ATCC 13979	4	99%
11	*Yersinia pseudotuberculosis*	ATCC 6905	2b	99%
12	*Yersinia intermedia*	ATCC 33645		NA
13	*Yersinia frederiksenii*	ATCC 29912		NA
14	*Yersinia aldovae*	ATCC 35236	Type strain	NA
15	*Yersinia aleksiciae*	Y159; CCUG 52872; DSM 14987; LMG 22254; WA758		NA
16	*Yersinia bercovieri*	ATCC 43970; CDC 2475-87; CNY 7506; WAIP 208; DSM 18528; CIP 103323; CCUG 26329		NA
17	*Yersinia kristensenii*	ATCC 29911; CDC YE1474		NA
18	*Yersinia mollaretti*	ATCC 43969; CDC 2465-87; CNY 7263; WAIP 204; CCUG 26331; CIP 103324; DSM 18520		NA
19	*Yersinia rohdei*	ATCC 43380; CDC 3022-85; CIP 103163; DSM 18270; LMG 8454; CCUG 38833; Aleksic H271/78		
20	*Yersinia ruckeri*	ATCC 29473; CDC 2396-61; NCIMB 2194; CCM 6093; CCUG 14190; CIP 82.80; DSM 18506; LMG 21879; NCIMB 2194; NCTC 12986		NA

### Phase 4: Environmental Background Panel

The 61 diverse environmental background organisms that were selected based on the recommendations of a panel of subject matter experts are listed in Table 3.^25^ Each organism was inoculated onto optimal solid medium and incubated under appropriate conditions for 24 to 48 hours. A single, isolated colony was selected and inoculated onto a second plate and incubated for 1 to 6 days, depending on the organism and its growth conditions. Plates were then sealed with parafilm, coded, and stored at 4°C until use. For testing, several colonies were removed and suspended in 4 mL BTA buffer to a final concentration of 10^9^ to 10^10^ CFU/mL (4 logs above LOD) and 0.150 mL added to the sample wells of 5 Plague LFAs.

**Table 3.** Environmental background panel

S. No.	Organism	Strain Name
1	*Acinetobacter calcoaceticus*	ATCC 14987; HO-1; NBRC 12552; NCIMB 9205; CIP 66.33; DSM 1139; LMG 1056
2	*Acinetobacter haemolyticus*	ATCC 17906; NCTC 10305; 2446/60; DSM 6962; CIP 64.3; NCIMB 12458
3	*Acinetobacter radioresistens*	ATCC 43998; DSM 6976; FO-1; CIP 103788; LMG 10613; NCIMB 12753
4	*Aeromonas veronii*	ATCC 35622; CDC 140-84
5	*Bacillus cohnii*	ATCC 51227; DSM 6307; LMG 16678
6	*Bacillus horikoshii*	ATCC 700161; DSM 8719; JP277; PN-121; LMG 17946
7	*Bacillus macroides (aka Lineola longa; Bacillus sp.)*	ATCC 12905; 1741-1b; DSM 54; NCIB 8796; NCIM 2596; NCIM 2812; LMG 18474
8	*Bacillus megaterium*	ATCC 14581; 7051; CCUG 1817, CIP 66.20, DSM 32, LMG 7127, NCIB 9376, NCTC 10342, NRRL B-14308
9	*Bacteroides fragilis*	ATCC 23745; ICPB 3498, NCTC l0581
10	*Brevundimonas diminuta*	ATCC 11568; DSM 7234; CCUG 1427, CIP 63.27, LMG 2089, NCIB 9393, NCTC 8545, NRRL B-1496, USCC 1337
11	*Brevundimonas vesicularis*	ATCC 11426; CCUG 2032, LMG 2350, NCTC 10900
12	*Burkholderia cepacian*	ATCC BAA-245; KC1766; LMG 16656; J2315; CCUG 48434; NCTC 13227
13	*Burkholderia stabilis*	2008724195; LMG 14294; CCUG 34168, CIP 106845, NCTC 13011; ATCC BAA-67
14	*Chromobacterium violaceum*	ATCC 12472; NCIMB 9131; NCTC 9757; CIP 103350; DSM 30191; LMG 1267
15	*Chryseobacterium gleum*	ATCC 29896; CDC 3531; NCTC 10795; LMG 12451; CCUG 22176; CDC 3531
16	*Chryseobacterium indologenes*	ATCC 29897; CDC 3716; NCTC 10796; CCUG 14483; CIP 101026; LMG 8337
17	*Citrobacter brakii*	ATCC 10053
18	*Citrobacter farmeri*	ATCC 31897; FERM-P 5539; AST 108-1
19	*Clostridium butyricum*	CDC 11875; ATCC 19398; NCTC 7423; VPI 3266; CCUG 4217; CIP 103309; DSM 10702; LMG 1217; NCIMB 7423
20	*Clostridium perfringens*	ATCC 12915; NCTC 8359; 3702/49; CIP 106516
21	*Clostridium sardiniense*	ATCC 33455; VPI 2971; DSM 2632; BCRC 14530
22	*Comamonas testosterone*	ATCC 11996; 567201; FHP 1343; NCIMB 8955; CIP 59.24; NCTC 10698; NRRL B-2611; DSM 50244; LMG 1800; CCUG 1426
23	*Deinococcus radiodurans*	ATCC 35073; NCIMB 13156; UWO 298
24	*Delftia acidovorans*	ATCC 9355; LMG 1801; CCUG 1822; CIP 64.36; NCIMB 9153; NRRL B-783
25	*Dermabacter hominis*	ATCC 49369; DSM 7083; NCIMB 13131; CIP 105144; CCUG 32998; S69
26	*Enterobacter aerogenes*	ATCC 13048; CDC 819-56; NCTC 10006; DSM 30053; CIP 60.86; LMG 2094; NCIMB 10102
27	*Enterobacter cloacae*	ATCC 10699; NCIMB 8151; CCM 1903
28	*Enterococcus faecalis*	ATCC 10100; NCIMB 8644; P-60
29	*Escherichia coli O157:H7*	ATCC 43895; CDC EDL 933; CIP 106327; O157:H7
30	*Flavobacterium mizutaii*	ATCC 33299; CIP 101122; CCUG 15907; LMG 8340; NCTC 12149; DSM 11724; NCIMB 13409
31	*Fusobacterium nucleatum subsp. Nucleatum*	ATCC 25586; CCUG 32989; CIP 101130; DSM 15643; LMG 13131
32	*Jonesia denitrificans*	ATCC 14870; CIP 55.134; NCTC 10816; DSM 20603; CCUG 15532
33	*Klebsiella oxytoca*	ATCC 12833; FDA PCI 114; NCDC 413-68; NCDC 4547-63
34	*Klebsiella pneumonia subsp. Pneumonia*	ATCC 10031; FDA PCI 602; CDC 401-68; CIP 53.153; DSM 681; NCIMB 9111; NCTC 7427; LMG 3164
35	*Kluyvera ascorbate*	ATCC 14236; CDC 2567-61; CDC 0408-78; DSM 30109; CCUG 21164; CIP 79.53
36	*Kluyvera cryocrescens*	ATCC 14237; CDC 2568-61; CCUG 544; NCIMB 9139; NCTC 10484
37	*Kocuria kristinae*	ATCC 27570; DSM 20032; NRRL B-14835; CCUG 33026; CIP 81.69; LMG 14215; NCTC 11038
38	*Lactobacillus plantarum*	ATCC BAA-793; LMG 9211; NCIMB 8826
39	*Listeria monocytogenes*	ATCC 7302; BCRC 15329
40	*Microbacterium sp.*	ATCC 15283; MC 100
41	*Micrococcus lylae*	ATCC 27566; CCUG 33027; DSM 20315; NCTC 11037; CIP 81.70; LMG 14218
42	*Moraxella nonliquefaciens*	ATCC 17953; NCDC KC 770; NCTC 7784; CCUG 4863; LMG 1010; BCRC 11071
43	*Moraxella osloensis*	ATCC 10973; CDC Baumann D-10; LMG 987; CCUG 34420
44	*Myroides odoratus*	ATCC 29979; NCTC 11179; LMG 4028; DSM 2802; CIP 105169
45	*Mycobacterium smegmatis*	ATCC 20; NCCB 29027
46	*Neisseria lactamica*	ATCC 23970; CDC A 7515; CCUG 5853; CIP 72.17; DSM 4691; NCTC 10617
47	*Pseudomonas aeruginosa*	ATCC 15442; NRRL B-3509; CCUG 2080; DSM 939; CIP 103467; NCIMB 10421
48	*Pseudomonas fluorescens*	ATCC 13525; Migula biotype A; NCTC 10038; DSM 50090; NCIMB 9046; NRRL B-2641; LMG 1794; CIP 69.13; CCUG 1253
49	*Ralstonia pickettii*	ATCC 27511; CCUG 3318; LMG 5942; CIP 73.23; NCTC 11149; DSM 6297; NCIMB 13142; UCLA K-288
50	*Rhodobacter sphaeroides*	ATCC 17024; ATH 2.4.2
51	*Riemerella anatipestifer*	ATCC 11845; CCUG 14215; LMG 11054; MCCM 00568; NCTC 11014; DSM 15868
52	*Shewanella haliotis (Pseudomonas putrefaciens)*	ATCC 49138; AmMS 201; ACM 4733
53	*Shigella dysenteriae*	ATCC 12039; CDC A-2050-52; NCTC 9351
54	*Sphingobacterium multivorum*	ATCC 33613; CDC B5533; NCTC 11343; GIFU 1347
55	*Sphingobacterium spiritivorum*	ATCC 33300; DSM 2582; LMG 8348
56	*Staphylococcus aureus* subsp*. Aureus*	ATCC 700699; CIP 106414; Mu 50, MRSA
57	*Staphylococcus capitis*	ATCC 146; NRRL B-2616; BCRC 15248
58	*Stenotrophomonas maltophilia*	ATCC 13637; NCIMB 9203; NCTC 10257; NRC 729; CIP 60.77; DSM 50170; LMG 958; NRRL B-2756
59	*Streptococcus equinus*	ATCC 15351; 7H4; NBRC 12057; IFO 12057
60	*Streptomyces coelicolor*	ATCC 10147; DSM 41007; NIHJ 147; NBRC 3176
61	*Vibrio cholerae*	ATCC 14104; BG29

### Phase 5a: White Powder Panel

A stakeholder panel consisting of representatives from state public health laboratories, CDC, DOD, EPA, the FBI, and the commercial sector identified 26 white powders (Table 4) that were commonly encountered by first responders and CDC Laboratory Response Network (LRN) reference laboratories.^25,26^ These powders were evaluated for their ability to affect the performance of the assay. Ten milligrams (10 mg) of each powder was suspended in 1.00 mL of BTA buffer (final concentration 10 mg/mL) and vortexed for 10 seconds. The suspension was allowed to settle for at least 5 minutes before 0.150 mL aliquots of the supernatant was removed and added to the sample wells of 5 Plague LFAs.

**Table 4.** White Powder Panel

S.No.	Material	Source
1	Dipel (*Bacillus thuringiensis*)	Summerwinds Nursery, Palo Alto, VA
2	Powdered milk	Raley's Grocery Store, Pleasanton, CA
3	Powdered coffee creamer	Raley's Grocery Store, Pleasanton, CA
4	Powdered sugar	Raley's Grocery Store, Pleasanton, CA
5	Talcum powder	Raley's Grocery Store, Pleasanton, CA
6	Wheat flour	Van's, Livermore, CA
7	Soy flour	Van's, Livermore, CA
8	Rice flour	Ranch 99, Pleasanton, CA
9	Baking soda	Target Stores, Livermore, CA
10	Chalk dust	Target Stores, Livermore, CA
11	Brewer's yeast	GNC Stores, Livermore, CA
12	Drywall dust	Home Depot, Livermore, CA
13	Cornstarch	Raley's Grocery Store, Pleasanton, CA
14	Baking powder	Raley's Grocery Store, Pleasanton, CA
15	GABA (Gama aminobutyric acid)	Sigma-Aldrich Corp, St. Louis, MO
16	L-Glutamic acid	Sigma-Aldrich Corp, St. Louis, MO
17	Kaolin	Sigma-Aldrich Corp, St. Louis, MO
18	Chitin	Sigma-Aldrich Corp, St. Louis, MO
19	Chitosan	Sigma-Aldrich Corp, St. Louis, MO
20	Magnesium sulfate (MgSO4)	Sigma-Aldrich Corp, St. Louis, MO
21	Boric acid	Sigma-Aldrich Corp, St. Louis, MO
22	Powdered toothpaste	Walmart Pharmacy, Livermore, CA
23	Popcorn salt	Raley's Grocery Store, Pleasanton, CA
24	Baby powder	Target Stores, Livermore, CA
25	Powdered infant formula, iron fortified	Target Stores, Livermore, CA
26	Powdered infant formula, low iron	Target Stores, Livermore, CA

### Phase 5b: White Powder Spiked with *Y. pestis* strain A1122

The 26 white powders tested in Phase 5a were spiked with *Y. pestis* strain A1122 and tested to determine if the white powders affected detection of *Y. pestis* by the LFA. Ten milligrams of each white powder were suspended in 0.900 mL of BTA buffer, and 0.100 mL of a suspension of *Y. pestis* strain A1122 was added to a final concentration of 10^6^ to 10^7^ CFU/mL (1 log above the LOD). Each tube was vortexed for 10 seconds. The suspensions were allowed to settle for at least 5 minutes; then 0.150 mL aliquots of the supernatant were removed and added to the sample wells of 5 LFAs to understand the degree, if any, to which the powder inhibited detection of *Y. pestis*.

### Phase 6a: Environmental Filter Extract

A pooled aerosol filter extract (from 30 different filters), which contained 6 μg protein/μL, was prepared as previously described^25^ and shipped to the test site. Operators added 1.00 mL BTA buffer to 1.00 mL extract. After mixing for 10 seconds, the suspension was allowed to settle for at least 5 minutes, and 0.150 mL aliquots of supernatant (450 μg total protein) were added to the sample wells of 5 Plague LFAs.

### Phase 6b: Environmental Filter Extract Spiked with *Y. pestis*

The pooled aerosol filter extract was spiked with *Y. pestis* strain A1122, at a final concentration of 10^6^ to 10^7^ CFU/mL, and retested. After mixing for 10 seconds, the suspension was allowed to settle for at least 5 minutes, followed by removal of 0.150 mL aliquots of the supernatant for testing. The spiked pooled filter extract was tested a total of 5 times.

### Phase 7: Temperature Dependent Expression of F1 Capsular Antigen

*Y. pestis* strains C099-3015 (Orientalis), Nepal 516 (Antiqua), and PyH1R3 (Mediaevalis), Pestoides A (Pestoides) were grown to test detection of temperature-dependent expression of *Y. pestis* F1 capsular antigen. Strains were inoculated onto 6% SBA and incubated either at 25°C or 35°C for 24 hours. Plates were incubated at 35°C for 24 hours followed by 25°C for an additional 4 days prior to testing. Cell suspensions were prepared in 2 mL of 0.85% sterile saline, lightly vortexed for homogenization, and adjusted to an absorbance of 1.0 (5.5 x 10^9^ CFU/mL) as previously described. Suspensions for testing were prepared by diluting 10-fold in BTA buffer and lightly vortexing immediately prior to testing. A concentration of 1 above the LOD was tested in 20 replicates at each temperature for each strain.

### Statistical Analysis

Titration curves, Receiver Operator Characteristic Curves (ROC) based on BTA Reader values were made using GraphPad Prism version 7.04 for Windows (GraphPad Software, La Jolla, CA, www.graphpad.com). Plague BTA test values were used for generating the interactive dot plots of LFA sensitivity and specificity calculations, and assay performance evaluation using MedCalc Statistical Software version 18.11.3 (MedCalc Software bvba, Ostend, Belgium; https://www.medcalc.org, 2019).

## Results

A multiphase study was conducted to evaluate and assess the performance of the Plague BTA LFA; 0.15 mL of BTA buffer was used as negative control, and 0.15 mL of *Y. pestis* strain A1122 at a concentration of 10^6^-10^7^ CFU/mL was used as a positive control in each phase of the study. A total number of 1,182 LFA tests were performed in this study, and details about positive and negative controls tested and number of samples tested in each phase of the evaluation are shown in Table 5.

**Table 5.** Details of the number of samples tested, including the positive and negative controls by plague BTA LFA test in each of the 7 phases

Yp LFA Test Phases	Positive Control	Negative Control	Positive Test Result	Negative Test Result	Total
Phase 1: Linear dynamic range and reproducibility testing	5	11	164	21	201
Phase 2: Inclusivity panel testing	5	5	80	5	95
Phase 3: Near-neighbor panel testing	5	5	0	100	110
Phase 4: Environmental background microbes testing	5	5	0	305	315
Phase 5a: White powder panel testing	10	5	0	130	145
Phase 5b: White powder panel spike testing	5	5	130	0	140
Phase 6: Environmental aerosol filter extract testing	3	3	5	5	16
Phase 7: Temperature dependent expression of F1 capsular antigen panel testing	0	0	80	80	160
Total	38	39	459	646	1,182

In the Phase 1 range finding and reproducibility study, a total of 185 tests were performed: 35 tests for range-finding of strain CO99-3015, 30 tests for range-finding of strain A1122, and 120 tests for reproducibility LOD testing of strain CO99-3015, along with positive and negative controls. All samples tested at a concentration >10^5^ to 10^6^ CFU/mL were positive. In Phase 2 inclusivity panel evaluation, a total of 90 tests (5 replicates of 18 different *Y. pestis* strains) were tested. Near neighbors of *Y. pestis* were evaluated in Phase 3, and a total of 100 tests (5 replicates of 20 near neighbor strains along with positive and negative controls) were performed. In the Phase 4 environmental panel evaluation, a total of 305 tests (5 replicates of 61 different bacterial strains) were tested. In the Phase 5 white powder and environmental aerosol sample test evaluation, a combined total of 260 tests (5 replicates of 26 white powders) in the absence and presence of *Y. pestis* A1122 were performed. In Phase 6, BioWatch aerosol filter extract was tested in 5 replicates each, with and without the spiking of *Y. pestis* A1122. In phase 7, the temperature-dependent detection of F1 capsular antigen was evaluated with a total of 160 tests, 20 replicates each of 4 different strains grown at 25°C or 35°C.

**Figure 1.**
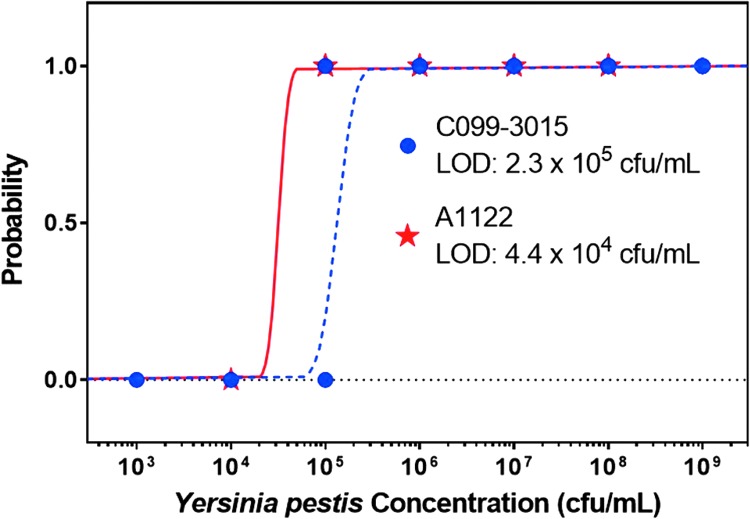
Probit regressions for the *Y. pestis* strains C099-3015 and A1122 strains are shown as 2 different lines in the scatter plot. The curves are drawn using the calculated probability of detection as a function of spore concentration. Limit of detection of the Yp LFA test was estimated by finding the *Y. pestis* strain concentration with a probability of detection at 0.95. For *Y. pestis* C099-3015, the LOD is 2.3 x 10^5^ CFU/mL, and for *Y. pestis* A1122 the LOD is 4.4 x 10^4^ CFU/mL.

Data from Phase 1 range-finding study, 35 tests (5 replicates for each of 7 different concentrations) of *Yp* strain CO99-3015 and 30 tests (6 replicates of 5 different concentrations) of *Yp* strain A1122 were used in the Probit regression analysis for determining the LOD of Plague BTA LFA visual results. LOD was calculated as the concentration that corresponds to a probability of 0.95, which is equivalent to the estimated LOD within 95% confidence intervals.^27^ Probit analysis curves are shown in Figure 1 for estimating the LOD for *Y. pestis* strains CO99-3015 and A1122. The calculated LOD based on Phase 1 data and Probit analysis for *Y. pestis* C099-3015 was 2.3 x 10^5^ CFU/mL (3.4 x 10^4^ CFU/assay), and for *Y. pestis* A1122, it was 4.4 x 10^4^ CFU/mL (6.6 x 10^3^ CFU/assay).

**Figure 2.**
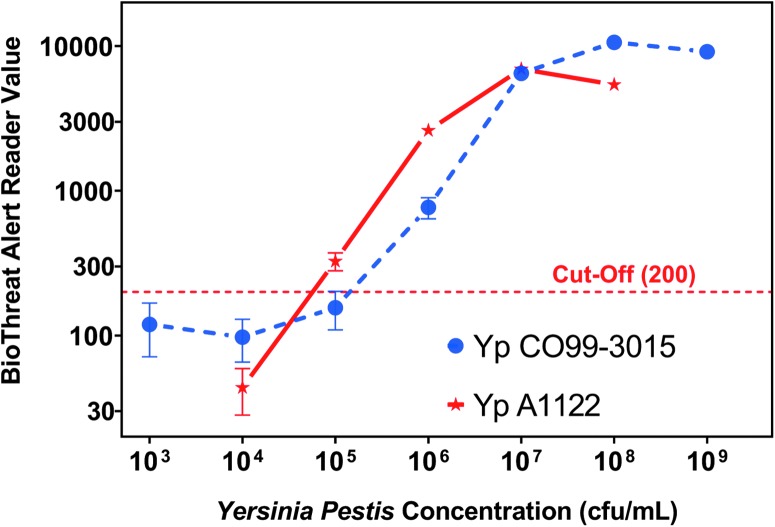
The titration curves depict BTA reader value with respect to the concentration of *Y. pestis* strains C099-3015 and A1122. The curves were generated using the average of at least 5 replicates, and the error bars are the standard deviations. The cut-off value of 200 is shown as a dashed line. For both strains, the first test concentration that is above the cut-off value is 10^5^ CFU/mL.

BTA Reader values were plotted against various concentrations of *Y. pestis* for determining the limit of detection as shown in Figure 2. The curves show that 10^5^-10^6^ CFU/mL uniformly gave positive results above the BTA Reader cut-off value of 200 and determined as the LOD.

The Plague LFA assay was further tested in Phase 1 for repeatability by 2 operators, using a final concentration of 10^5^-10^6^ for a total of 120 tests using *Y. pestis* strain CO99-3015, of which 120 of 120 tests gave expected positive results (both by visual observation and by BTA Reader call).

In Phase 2, an inclusivity panel of 18 *Y. pestis* strains was tested 5 times each by 1 of 2 different operators (Table 1). The inclusivity panel consisted of 17 F1 capsular antigen positive strains and one F1 capsular antigen minus strain, Angola O.PE3. The 17 F1 capsular antigen positive *Y. pestis* strains yielded 85 positive visual results and 84 of 85 BTA Reader positives. The 1 BTA Reader negative plague LFA was re-read on a second reader and was positive. The 1 F1 capsular antigen minus strain yielded 5 negative visual results and 5 negative BTA Reader results.

In Phase 3, a near neighbor panel of 20 *Yersinia* (non-*pestis*) strains (Table 2) was tested by 5 different operators at a concentration of 10^8^ to 10^9^ CFU/mL (3 logs above the LOD). All tests yielded negative results both visually and by BTA Reader. In Phase 4, an environmental panel of 61 strains (Table 3) yielded negative test results for 58 of 61 (95%) strains by visual observation and using BTA Reader. Positive plague LFA results were observed with *Brevundimonas diminuta*, *Myroides odoratus*, and *Staphylococcus aureus.* In Phase 5, the white powders did not affect the performance of the LFA as evidenced by the presence of the positive control line in 130 of 130 tested samples and the absence of any false-positive result in the test line. When the powders were spiked with *Y. pestis* strain A1122, positive results were observed with 125 of 130 tests. Inhibition (5 of 130 tests) of detection of *Y. pestis* strain A1122 was observed with only chalk dust and drywall dust. In Phase 6, the environmental filter extract did not inhibit the performance of the LFA, as evidenced by the presence of a positive control line in 5 of 5 tested samples without false-positive result. Filter extract spiked with *Y. pestis* strain A1122 yielded positive results in all 5 replicates.

**Figure 3.**
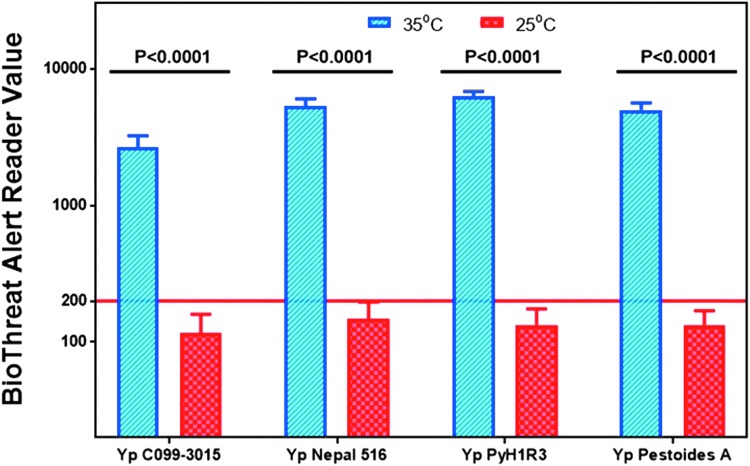
Bar diagram that summarizes the testing performed on *Y. pestis* strains Yp, C099-3015 (Orientalis); Yp, Nepal 516 (Antiqua); Yp, PyH1R3 (Mediaevalis); Yp, Pestoides A (Pestoides) were grown either at 25°C or at 35°C, or 24 hours followed by 25°C for 4 additional days. It provides a visual representation of the BTA Reader values in 2 different growth temperatures. The number of tests performed per sample are displayed at the top of each cluster. The cutoff value of 200 is shown as a solid line. Any data points that were above the cut-off value are positive, while any data points below the cut-off value are negative.

The Plague LFA assay was also evaluated in Phase 7 for its ability to detect temperature-dependent expression of *Y. pestis* F1 capsular antigen. Four *Y. pestis* strains representing each of the biovars C099-3015 (Orientalis), Nepal 516 (Antiqua), PyH1R3 (Mediaevalis), and Pestoides A (Pestoides), were tested 20 times each for the 2 growth conditions (a total of 160 tests were performed). Strains grown at 25°C resulted in 80 of 80 visual negative results, and 73 of 80 negative BTA reads. The 7 positive LFA strips were re-read on a second BTA reader and were negative. Strains grown at 35°C for 24 hours followed by 25°C for 4 days resulted in 100% positive results both visually and by the BTA Reader. The effect of growth temperature on detection was statistically significant (*P* < 0.0001) (Figure 3).

Excluding the 33 positive controls and 39 negative controls performed during the evaluation, the data from the 1,110 tests performed during Phases 1 through 7 were used to calculate sensitivity and specificity. Sensitivity, specificity, and accuracy are basic measures of performance for a diagnostic/detection test. Together, they describe how well the test can determine whether the *Y. pestis* F1 antigen (the analyte) is present or absent in the tested sample. Data from visual reads of the plague BTA LFA are displayed in a 2 x 2 contingency table format (Table 6). Test results fall in 1 of the 4 categories: true positive (TP, *Y. pestis* antigen present, and test positive); false positive (FP, *Y. pestis* antigen not present but test positive); false negative (FN, *Y. pestis* antigen present but test negative), and true negative (*Y. pestis* antigen absent and test negative). Of the 1,110 tests performed, 460 were true positive, 625 were true negative, 15 were false positive, and 10 were false negative.

**Table 6.** 2 x 2 Contingency table to assess the accuracy of *Y. pestis* LFA by visual read

Plague BTA LFA	Y. pestis Positive	Y. pestis Negative	Total
Test Positive	459	15	474
Test Negative	10	626	636
Total	469	641	**1,110**
Statistical analysis of *Y. pestis* LFA performance			
*Parameter*	*Percentage*		*Confidence Interval*
Sensitivity	97.868%		96.114% to 98.973%
Specificity	97.660%		96.170% to 98.684%
Area Under the Curve (AUC)	0.978		0.967 to 0.986
Accuracy	97.748%		

Sensitivity is defined as the proportion of true positives that are correctly identified by the test:

$$Sensitivity\left(\%\right)=100\times \frac{TP}{TP+FN}.$$

Specificity is defined as the proportion of true negatives that are correctly identified by the test and is calculated as:

$$Specificity\left(\%\right)=100\times \frac{TN}{TN+FP}.$$

Accuracy is defined as the proportion of the total number of true-positive and true-negative tests that are correctly identified by the test and is calculated as:

$$Accuracy\left(\%\right)=100\times \frac{TN+TP}{TP+FN+TN+FP}.$$

Data from only 1,110 *Yp* BTA LFA test results are used for calculations. Data from testing of 72 samples that were used as positive and negative controls were excluded in the diagnostic test sensitivity and specificity determination. Table 6 shows the 2 x 2 contingency table and statistical analysis results for the resulting sensitivity (97.868%), specificity (97.660%), and accuracy (97.748%) of this assay.

**Figure 4.**
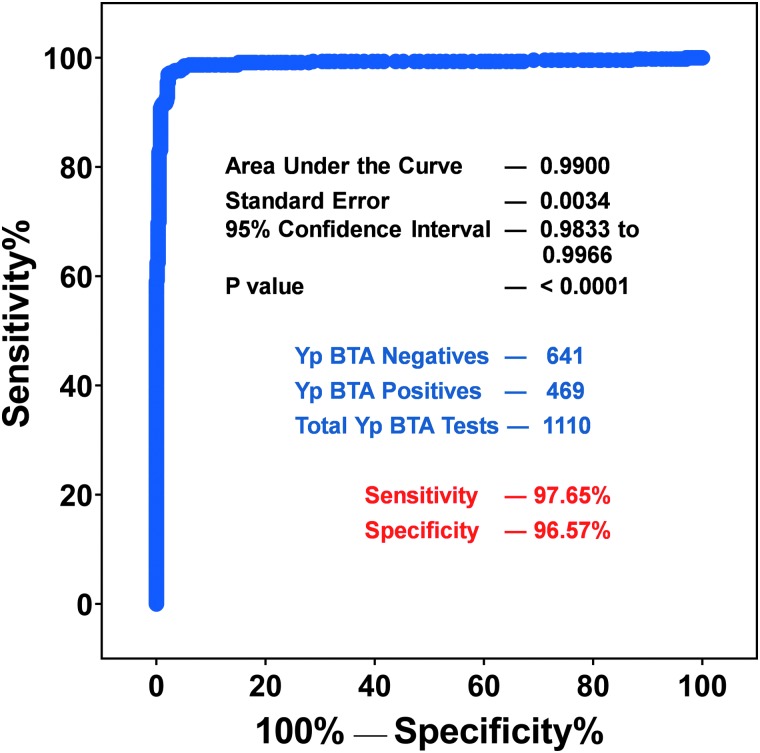
Receiver operator characteristic (ROC) curve provides a graphic representation of the sensitivity and specificity of the visual test results of *Yp* LFA test. Each point on the curve is a possible cut-off value, and its place on the curve is determined by its specificity and sensitivity. The calculated area under the curve (AUR) was 0.99, thus indicating that the assay is accurate and reliable.

To further evaluate the assay, the BTA Reader values were used to generate a Receiver Operating Characteristic (ROC) curve. For Phases 1, 2, and 7, the BTA Reader values used were from the rerun on the second reader. Even though the reader values are not quantitative, the values can be used to further evaluate the accuracy of a detection test to discriminate the test positive samples from those that are test negative using ROC analysis. The sensitivity and specificity were calculated for every possible cut-off point selected to discriminate between the positive and negative populations. This curve was created by plotting the true-positive rate as a function of the false-negative rate for every possible cut-off point. Figure 4 shows the ROC curve for the Plague LFA, having the area under the curve 0.99, thus indicating that this test is highly specific and sensitive. The sensitivity (97.65%) and specificity (96.57%) calculated from the ROC curve are based on a BTA cut-off value of 200. These values are lower than the calculated sensitivity (97.868%) and specificity (97.660%) shown using visual plague BTA LFA results (Table 6). The Youden index J is the maximum vertical distance between the ROC curve and the line of equality. The cutoff value that responds to the Youden index J can give the optimal combination of sensitivity and specificity, if the disease prevalence is 50%. The Youden index J calculated from this ROC curve was 0.9483, and the calculated best sensitivity of 97.01% and specificity of 97.82% at a cut-off of BTA Reader value >238. When reader value is available, different cut-offs can be set to calculate the sensitivity and specificity of the assay.

**Figure 5.**
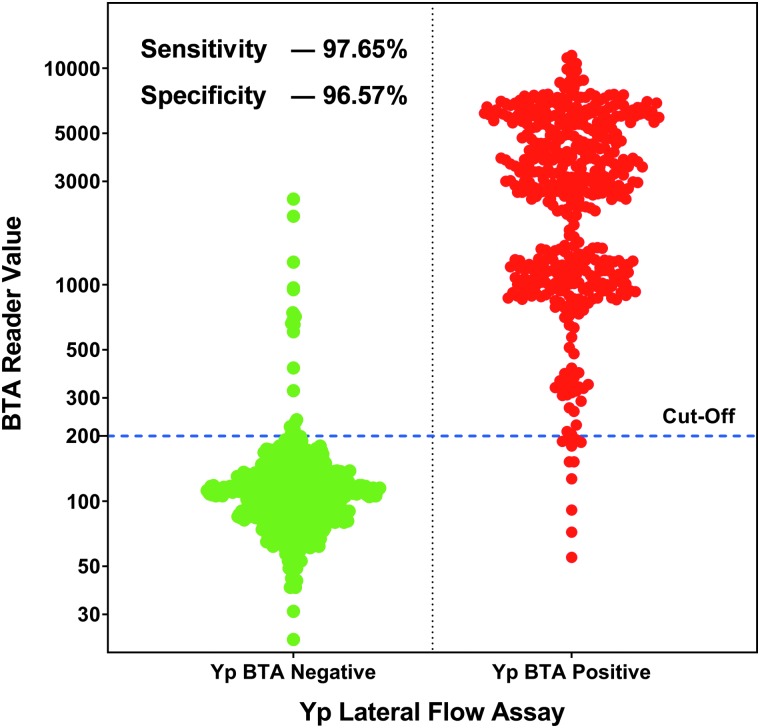
A dot density diagram that shows all 1,110 tests performed grouped as designated positive and designated negative by the BTA Reader. The cut-value of 200 is shown as a solid line. The calculated assay sensitivity is 97.65%, and the specificity is 96.57%. Any data points in the designated negative group that were above the cut-off value are false positive, while any data points in the designated positive group that were below the cut-off value are false negative.

In addition, data required for ROC analysis can also be depicted as an interactive dot plot (Figure 5) for measuring the sensitivity and specificity. In this plot, the reader values are shown on the Y axis, and different cut-off values can be used to estimate the sensitivity and specificity at that value. In this analysis, a threshold reader value of 200 also gave a sensitivity of 97.65% and specificity of 96.57%.

## Discussion

In addition to natural outbreaks (ie, the 3 major pandemics), *Y. pestis* has been employed and/or developed as a biological weapon. During World War II, Japan's infamous Unit 731 purportedly dropped plague-infected fleas over Manchuria.^4^ Both the United States and the former Soviet Union investigated the use of *Y. pestis* as a weapon, including methods of aerosolization allowing direct dispersal of the bacteria.^4,7,14^

An effective public health response to *Y. pestis* involves several facets, including use of medical countermeasures (vaccines, antimicrobials) and methods for surveillance and early diagnosis. Because of the high mortality rates associated with untreated pneumonic plague, rapid identification of a deliberate release is critical so that medical countermeasures can be rapidly deployed and potentially exposed individuals can be treated. Currently, many technologies exist for the detection and identification of *Y. pestis*. Polymerase chain reaction (PCR) assays and platforms, including the RAPID and SmartCycler, have demonstrated sensitivities of 50 fg DNA (∼10 genomic equivalents, GE) for single-probe assays,^28^ while the FilmArray detected *Y. pestis* strains at 250 to 25,000 GE.^29^ Yang et al^30^ investigated the use of 2 suspension arrays for the detection of *Y. pestis*, one of which could identify only to the genus level. The species-specific assay had a detection limit of 50 fg DNA. Fluorescence in situ hybridization has also been investigated but is impractical because of its lengthy assay time (∼8 hours to results).^32^ Most antibody-based assays target the F1 capsular protein.^33-36^

LFA assays were first commercially introduced for pregnancy testing in 1988.^37^ LFA assays require minimum sample and no specialized equipment^38^ and could be used by first responders and law enforcement officers to test suspicious materials in field settings. An F1-based lateral flow that used monoclonal antibodies to F1 has been developed and used in Madagascar to test bubo aspirates from suspected plague patients.^32^ F1 antigen is temperature regulated and not expressed at temperatures <33°C; as an alternative, a dipstick employing antibodies against the Pla protein is not limited by variations in growth temperature, although it is present in *Y. pseudotuberculosis* and *Y. enterocolitica,* and recent studies have demonstrated it is also found in other bacteria and therefore not a specific to *Y. pestis.*^33^ Anti-LPS antibodies have also been evaluated and may hold promise for species-specific detection.^34^

Other BTA LFA assays have previously been evaluated for the detection of biothreat agents, including orthopoxviruses,^39^ ricin,^40^ abrin,^41^ and *Bacillus anthracis.*^25^ Limited evaluations have also been conducted with assays for the detection of *Francisella tularensis* (unpublished data), botulinum neurotoxins,^42^ and staphylococcal enterotoxins.^43^ The Plague BTA LFA test uses a combination of a polyclonal capture antibody and monoclonal detect antibody to selectively capture and detect the presence of F1 antigen in aqueous samples. The purpose of the current study was to evaluate the performance of the Plague LFA assay in order to understand its sensitivity, specificity, reproducibility, and limitations for potential use in the field. Using the BTA Reader and the manufacturer's recommended cutoff of 200, we estimated the LOD of the Plague LFA to be approximately 10^5^ to 10^6^ CFU/mL. This LOD is lower than reported in an earlier study, in which Zasada et al demonstrated an LOD of 10^7^ CFU/mL for *Y. pestis* using the Plague LFA assay. The difference in LOD may be because in the previous study, *Y. pestis* organisms were inactivated by heating at 60°C for 22 hours prior to testing with the Plague BTA Test Strip. Limitations of the Plague BTA LFA include the relatively high LOD compared to real-time PCR methods and the inability to detect *Y. pestis* grown at 25°C.

To evaluate the sensitivity and specificity of the Plague BTA LFA assay, 18 *Y. pestis* strains belonging to 4 different biovars and of different geographic origins were used. Seventeen of these strains yielded positive results visually when tested at a concentration 2 logs above the LOD. The Angola O.PE3 strain is an F1 capsular antigen minus strain and, as expected, was negative by this assay. The Plague LFA was also tested against 20 *Yersinia* near neighbors as well as 61 other organisms commonly encountered in the environment. Positive results were observed with environmental background strains; *B. diminuta*, *M. odoratus* (the F1 capsule antibody used in the Tetracore Plague LFA targets an antigenic epitope that is also present in *M. odoratus* and *B. diminuta* where there is an overlap in the F1 capsule antigen protein sequence from positions 103 to 119), and *S. aureus*. Antibodies used in the Plague BTA LFA are purified on a protein A column, and this protein is found in the cell walls of some organisms (eg, *S. aureus*).

In this study, we evaluated the ability of this assay to detect *Y. pestis* in the presence of commonly encountered powders and extracts taken from environmental filters. The Plague LFA yielded positive results in 24 of 26 powders spiked with *Y. pestis* A1122 at a final concentration of 10^6^-10^7^ CFU/mL. Inconsistent results were observed with chalk dust and drywall dust, both of which consist of relatively large particles that may inhibit the flow of fluid across the test strip and that may also act as a filter to prevent the antigen from interacting with the antibody, resulting in a false-negative result. Pooled environmental filter extracts alone yielded negative results and did not prevent the assay from detecting *Y. pestis* at a final concentration of 10^6^-10^7^ CFU/mL.

Variability in readings between BTA Readers was encountered, which yielded either false-positive or false-negative readings. In the case of false-positive results, the reader typically produced values near the cut-off. These findings highlight the importance of these assays being performed by trained and experienced users with an understanding of the limitations of sample testing and result interpretation.

In conclusion, the results presented here demonstrate a sensitivity (97.87%), specificity (97.66%), accuracy (97.75%), and limit of detection (10^4^-10^5^ CFU/ml) for the Plague BTA LFA. These performance data are important for accurate interpretation of qualitative results arising from testing suspicious white powders and aerosol samples in the field. It should be noted that specificity of this test has not been evaluated for other environmental specimens such as soil or water. It is not approved for human clinical diagnostic use. Highly suspicious samples should be tested by other methods in a reference laboratory. If only limited or very sparse material is available, it should be collected and submitted to a CDC LRN laboratory for testing. In addition, this assay is based on detection of *Y. pestis* F1 capsule, an antigen expressed only at ≥33°C. Any positive specimen in this assay is considered presumptive positive and should be referred to the CDC LRN for additional testing, confirmation, and characterization for an appropriate public health response.
